# Pulmonary sarcomatoid carcinoma: a clinicopathologic study and prognostic analysis of 51 cases

**DOI:** 10.1186/1477-7819-11-252

**Published:** 2013-10-02

**Authors:** Si-Yuan Huang, Shu-Jing Shen, Xing-Ya Li

**Affiliations:** 1 Department of Medicine Oncology, The First Affiliated Hospital of Zhengzhou University, 450052 No.1 of Jianshe east Road, Zhengzhou, China

**Keywords:** Pulmonary sarcomatoid carcinoma, Clinical characteristics, Cathological characteristics, Treatment, Prognosis

## Abstract

**Background:**

Pulmonary sarcomatoid carcinoma is a diagnostically challenging group of tumors. It’s a rare histologic subtype of non-small cell lung cancer.There are five subgroups of pulmonary sarcomatoid carcinoma, they are identified as pleomorphic carcinoma, spindle cell carcinoma, giant cell carcinoma, carcinosarcoma, and pulmonary blastoma. We explored the clinicopathologic features and prognostic factors of this tumor.

**Methods:**

We analyzed retrospectively the clinicopathological data of 51 patients with pulmonary sarcomatoid carcinoma who were treated in the First Affiliated Hospital of Zhengzhou University, Henan Cancer Hospital and Henan People Hospital from January 2005 to December 2012. The correlation between prognosis and age, sex, smoking history, tumor size, TNM staging, and treatment modality was analyzed by the statistical software SPSS 17.0. The survival analysis was conducted using the Kaplan-Meier method. The factors influencing survival were analyzed using univariate (Log-rank) and multivariate (Cox) models.

**Results:**

The overall survival rates at 1, 2, 3 and 5 years were 45.5%, 35.8%, 28.2% and 20.1%, respectively. Cox univariate analyses revealed that age, tumor size, T stage, M stage, surgery or not, and postoperative chemotherapy or not, were prognostic factors. Cox multivariate analysis found that tumor size and M stage were independent prognostic factors for PSC.

**Conclusions:**

Due to its rarity and the lack of large-scale clinical trial evidence, few studies about PSC have been reported, its clinical and pathological characteristics remain unclear, and its preoperative diagnosis and investigation of novel treatment approaches are imperative. In our study, the main factors affecting the prognosis of tumor size and M staging are the crucial prognostic factors for PSC. Surgical resection and postoperative adjuvant chemotherapy might result in better prognosis.

## Background

Sarcomatoid carcinoma can occur throughout the body; however, primary sarcomatoid carcinoma in the lung is very rare, accounting for 0.1% to 0.4% of all lung malignancies [1]. Pulmonary sarcomatoid carcinoma (PSC) is a rare histologic subtype of non-small cell lung cancer (NSCLC) [2]. PSC is defined as poorly differentiated non-small cell carcinoma that contains a component of sarcoma or sarcoma-like elements according to the 2004 World Health Organization [3].

PSC generally runs an aggressive clinical course and may cause major difficulties in the diagnosis; its rapid preoperative diagnosis is very difficult because of its heterogeneity [4]. The prognosis remains controversial. Although some insist that the prognosis of PSC is less favorable than other NSCLC among postoperative patients [1,5,6], others see no difference between PSC and other NSCLC [7,8].

This study discusses the clinicopathological features and prognostic factors for 51 cases of pulmonary sarcomatoid carcinoma. This retrospective analysis of clinical data, treatment methods and survival rates may yield clues to appropriate diagnostic and therapeutic strategies for this aggressive 'miniature monster’.

## Methods

Ethical approval: Any experimental research were performed with the approval of ethics committee. Research carried out on humans were in compliance with the Helsinki Declaration,

1. Patient inclusion criteria: The 51 patients with pulmonary sarcomatoid carcinoma treated in Henan Cancer Hospital, Henan People Hospital and First Affiliated Hospital of Zhengzhou University between January 2005 and December 2012 were included in this study. All specimens of these patients were confirmed by two experienced pathologists according to the 2004 WHO classification of PSC. The diagnosis was confirmed when at least 10% of the tumor contained a sarcomatoid component consisting of spindle or pleomorphic giant cells, or both.

2. Clinical data: In these 51 cases, five cases were carcinosarcoma, one case was pulmonary blastoma, five cases were spindle cell carcinoma, two cases were giant cell carcinoma, and the remaining cases were not diagnosed as a specific subtype of sarcomatoid carcinoma. Of these cases, 43 were males, 8 were females, and the male to female ratio was 5.4:1. Patients ranged in age from 30 to 80 years with a mean age of 57.8 years. A total of 18 patients had no history of smoking, 9 cases had a smoking history of <30 years, and 24 cases had a smoking history of ≥30 years. Incidence of cough, expectoration, chest pain, back pain and bloody sputum are in the majority in this group, and nine asymptomatic cases were detected via physical examination. For this group of patients, there were 15 cases involving the left upper lobe, 14 cases involving the lower lobe, 6 cases involving the right upper lobe, 4 cases involving the mid-lobe, and 3 cases involving the lower lobe. The remaining cases had multiple lesions. According to the international staging of lung cancer, the TNM stage of the 51 resection or biopsy pathologic results were as follows: 2 cases of Phase I (all Ia), 25 cases of Phase II (10 cases of IIa and 15 cases of IIb), 14 cases of Phase III (all IIIa), and 10 cases of Phase IV.

3. Follow-up: All patients were followed up (outpatient, telephone, or follow-up letter investigation) until 22 December 2012. Follow-up was 1 to 57 months, with median follow-up being 6 months. At the end of the observation, 31 patients had died and 20 patients had survived.

4. Statistical methods: Data were entered, analyzed, and processed using SPSS 17.0 statistical software. Data was conducted using the Kaplan-Meier method and draw the survival curve, Log rank method of prognostic factors in univariate analysis test, Cox model for multivariate analysis. The inspection level α = 0.05.

## Results

**Figure 1:**
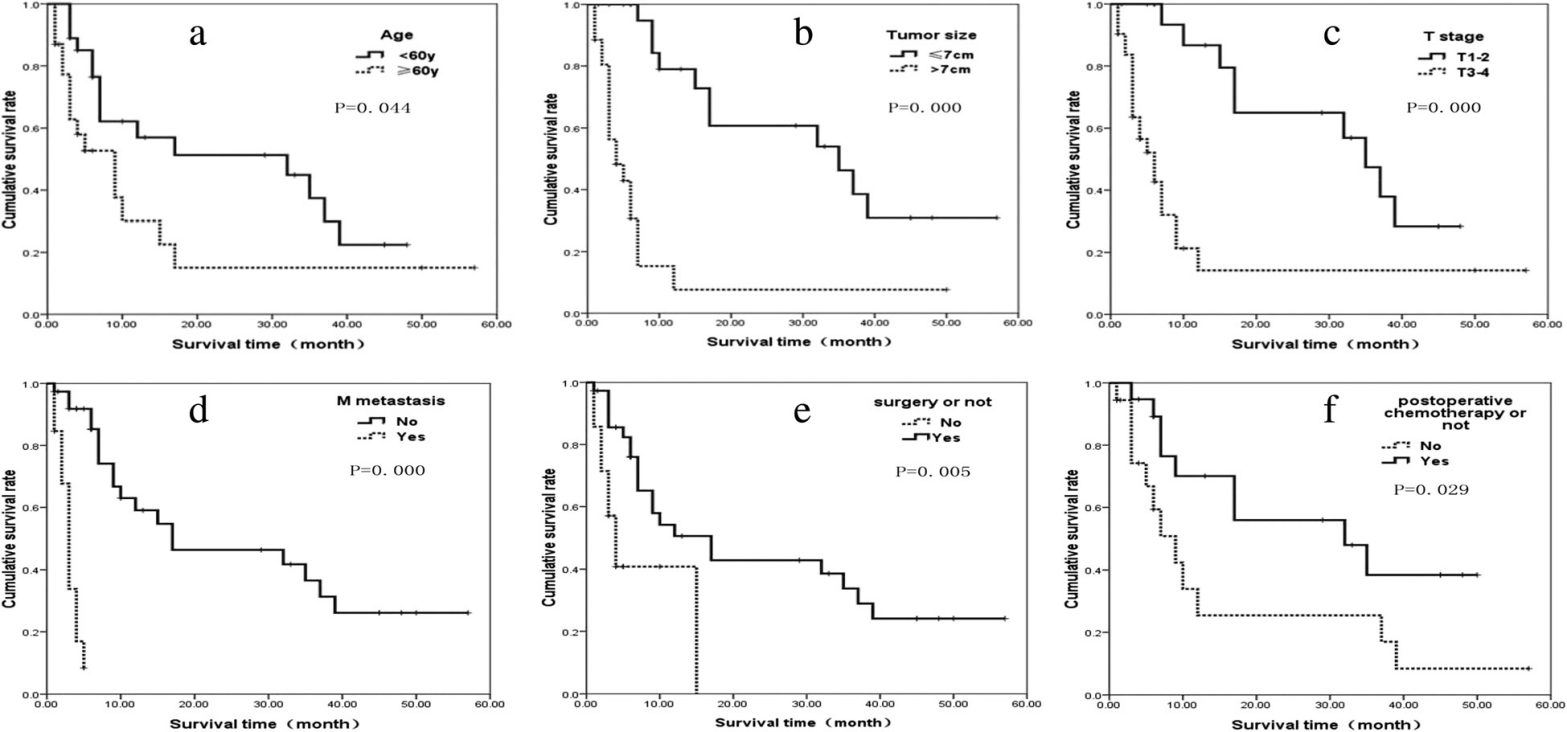
**The survival curve of 51 patients. a)** Different age patients with PSC survival curve comparison. **b)** Different tumor size patients with PSC survival curve comparison. **c)** Different T stage patients with PSC survival curve comparison. **d)** Different M stage patients with PSC survival curve comparison. **e)** Whether or not surgery PSC patients survival curves comparison. **f)** Whether postoperative chemotherapy in patients with PSC survival curve comparison. PSC, pulmonary sarcomatoid carcinoma.

1. Pathological results: Of the patients, 3 of 9 cases that were biopsied using a fiber endoscope were confirmed by pathological examination, and 12 of 18 cases were confirmed by computed tomography (CT)-guided lung tumor biopsy. The remaining 36 cases were not correctly diagnosed before surgery pathology results. Tumor size was calculated by the maximum diameter. The tumor sizes of this group were approximately 2 to 18.0 cm with an average size of 8 cm; 25 cases were ≤7 cm and 26 cases were >7 cm. Three of the 51 patients had neuroendocrine differentiation and one patient had large cell neuroendocrine carcinoma CD56 +.

2. Immunohistochemistry: Immunohistochemistry was performed using antibodies including Vim, CK, P63, S-100, Ki67, CK7, EMA, TTF-1, CK5/6, Syn etcetera by (streptavidin-perosidase( SP) method. Among those antibodies, CK and Vim showed the highest expression, 92.9% (39/42) and 90.5% (38/42), respectively; EMA showed a moderate positive, 90.0% (9/10); TTF-1, P63, CK5/6 and CK7 were positive at 33.3% (9/27), 43.8% (7/16), 42.3% (11/26) and 65.4% (17/26), respectively; and S-100 and Syn were expressed in the lowest amounts, 10.5% (2/19) and 20.0% (3/15), respectively.

3. Treatment methods: A total of 37 patients underwent ipsilateral lobectomy or pneumonectomy, combined with mediastinal lymph node dissection. After surgery, 19 cases received chemotherapy. The chemotherapy drugs used were gemcitabine, vinorelbine, paclitaxel, docetaxel, cisplatin and other routine chemotherapy drugs used for NSCLC.

4. Survival analysis: The average survival time of the patients was 13.3 months, and the median survival time was 6 months (95% confidence interval, 1.6 to 18.4 months). The 1-, 2-, 3- and 5-year overall survival rates were 45.5%, 35.8%, 28.2% and 20.1%, respectively. Statistical analysis was carried out on the clinical characteristics including gender, age, tumor size, smoking history and treatment factors and prognosis. The results are shown in Table 1.

5. Prognostic factors analysis results: Univariate analysis (Figure 1a-f) showed that prognosis of the patients was influenced by age, tumor size, T stage, metastatic status, surgery or not, and postoperative chemotherapy or not (*P* <0.05, Table 1). However, multivariate analysis showed that tumor size (*P* = 0.005) and metastatic status (*P* = 0.032) are independent prognostic factors (Table 2).

**Table 1:** **Clinicopathological characteristics and survival analysis of 51 patients with pulmonary sarcomatoid carcinoma** (**PSC)**

**Characters**	**Case numbers**	**The median survival time(month)**	**5-year survival rate (%)**	**x**^**2**^	***P***
Gender					
Male	43	6	21.7%	1.424	0.233
Female	8	4	21.4%		
Age					
≤60y	28	8.5	22.4%	4.055	0.044
>60y	23	4	15.1%		
Tumor size					
≤7 cm	25	29	30.8%	21.789	0.000
>7 cm	26	4	7.7%		
Smoking history					
No	18	5	25%	0.622	0.430
Yes	33	6	21.2%		
T stage					
T1-2	20	32	28.4%	12.423	0.000
T3-4	31	4	14.2%		
Lymph node metastasis					
No	27	7	24.2%	2.813	0.093
Yes	24	4	14.3%		
M metastasis					
No	38	9	26.1%	36.313	0.000
Yes	13	3	0		
Surgery or not					
Yes	37	7	24.1%	7.800	0.005
No	14	3.5	0		
Postoperative chemotherapy					
Yes	19	17	38.4%	4.755	0.029
No	18	5.5	8.5%		

**Table 2:** **Multivariate Cox model analysis of effects of** p**ulmonary sarcomatoid carcinoma** (**PSC) prognosis**

**Variable**	**Β**	**Wald**	**RR**	**95% CI**	***P***
Tumor size	1.369	7.743	3.931	1.499 to 10.310	0.005
M metastasis	1.803	4.622	6.067	1.173 to 31.388	0.032

## Discussion

PSC occurs mostly in men in their sixth and seventh decades, except for the pulmonary blastoma subtype, which occurs equally in men and women and frequently in the fourth decade [9,10]. It shows prevalence among male smokers, with an average age at presentation of 60 years and who have a history of moderate to heavy tobacco consumption [7,11,12]. In this group, the male to female ratio was 5.4:1 and 63.0% (29/46) of the patients have a history of smoking; however, the smoking history did not have a significant influence on the prognosis (*P* = 0.430).

Compared to other histologic subtypes, PSC behaves in an aggressive way. In the major published series of PSC, no specific signs or symptoms have been found when compared with other typical NSCLC [11-15]. The preoperative diagnosis of PSC is very difficult. Although PSC may sometimes be diagnosed on cytological preparations, surgical or biopsy specimens are necessary to obtain a definitive diagnosis because of the heterogeneity of PSC. In this group, the preoperative misdiagnosis rate was 70.6% (36/51). Someone raised [16], A modified vimentin histologic score (M-VHS) could be an effective diagnostic tool for this cancer, as to whether can be applied to clinical needs further large-scale studies. Some reports [10,16-18] show that positron emission tomography (PET) uptake is much higher in sarcomatoid carcinoma than in other NSCLC (*P* <0.0001), which means a PET scan may effectively identify PSC, but further confirmation still depends on pathological diagnosis.

The average survival of patients with PSC was 13.3 months in our study, which is slightly longer than the 11 months that is reported in other published series [12]. It is possible that multimodality therapeutic approach may also have contributed to a better prognosis for advanced patients. The 1-, 2-, 3- and 5-year overall survival rates in this group were 45.5%, 35.8%, 28.2% and 20.1%, respectively. Multivariate analysis of prognostic factors revealed that the main factors affecting prognosis are tumor size and M staging.

The treatment principle for pulmonary sarcomatoid carcinoma is similar with other non-small cell lung cancer. In this study, surgery or not (*P* = 0.005) and postoperative chemotherapy or not (*P* = 0.029) both have significant impact on prognosis on a statistical level. Early surgery is the preferred treatment for PSC, for which the postoperative adjuvant chemotherapy can be performed. Given the current status of epidermal growth factor receptor tyrosine kinase inhibitor (EGFR-TKI) in treatment of NSCLC, could be considered for treatment of PSC? Until now, innovative treatments such as monoclonal antibodies, which are target epidermal growth factor receptor mutations, have shown no definitive effect on the outcome [17-19]. It needs more large population clinical studies to prove whether EGFR-TKI can bring the Gospel for patients with PSC or not. Tsao AS and his colleague show that PDGFR-b has a higher expression and an increase of gene copy number in PSC compared with other NSCLC, which suggests that perhaps PDGFR-b would become a new therapeutic target [20]. These studies suggest a new research direction for PSC treatment.

The study presents several limitations that must be considered when interpreting the results. First, the population in this study was small because the disease is rare. Second, this study includes a wide variety of patients. Some patients underwent curative resection, while some did not because of advanced disease. Some patients underwent chemotherapy, while some did not because of economic conditions. Third, the study includes only eight female patients. Future studies with larger patient population must re-evaluate the influence of gender so that we can obtain statistical power.

## Conclusions

In summary, many patients are easily misdiagnosed before accepting surgery, so an accurate preoperative histological diagnosis is imperative. Limited experience in diagnosis, treatment and prognosis of PSC still exists because the disease is rare and because there is a lack of large-scale clinical trial evidence. Because further research in diagnosis and treatment is urgent, we hope that the reports about PSC will gradually increase in order to facilitate clinical doctors to manage the disease and benefit the patients.

### Consent

Written informed consent was obtained from the patient for the publication of this report and any accompanying images.

## Abbreviations

CT: Computed tomography; NSCLC: Non-small cell lung cancer; PET: Positron emission tomography; PSC: Pulmonary sarcomatoid carcinoma; SP: Streptavidin-perosidase; M-VHS: A modified vimentin histologic score; EGFR-TKI: Epidermal growth factor receptor tyrosine kinase inhibitor.

## Competing interests

The authors declare that they have no competing interests.

## Authors’ contributions

All authors read and approved the final manuscript.
